# Establishment of a Rapid and Convenient Fluoroimmunoassay Platform Using Antibodies Against PDL1 and HER2

**DOI:** 10.3390/cimb47010062

**Published:** 2025-01-17

**Authors:** Ji Eun Choi, Hanool Yun, Hee-Jin Jeong

**Affiliations:** 1 Department of Pathology, Chungnam National University Sejong Hospital, Sejong 30099, Republic of Korea; 2 Department of Biological and Chemical Engineering, Hongik University, Sejong 30016, Republic of Korea

**Keywords:** fluorescence-linked immunosorbent assay, immunoassay, fluorescent biosensor, PDL1, HER2

## Abstract

The development of accurate and high-throughput tools for cancer biomarker detection is crucial for the diagnosis, monitoring, and treatment of diseases. In this study, we developed a simple and rapid fluorescence-linked immunosorbent assay (FLISA) using fluorescent dye-conjugated antibody fragments against programmed cell death ligand 1 (PDL1) and human epithelial growth factor receptor 2 (HER2). We optimized key steps in the FLISA process, including antigen immobilization, blocking, and antibody reaction, reading the assay time to 3 h—significantly faster compared to the 23 h duration of usual FLISA. The limit of detection for the rapid FLISA in detecting PDL1 was lower than that of FLISA, and the detection of HER2 was similar between the two methods, indicating that the rapid FLISA provides a fast and accurate approach for detecting PDL1 and HER2. This robust platform can be readily adapted for various fluoroimmunoassays targeting other antigens of interest.

## 1. Introduction

Rapid, high-throughput, and accurate detection of cancer biomarkers is essential for early diagnosis and disease monitoring. Rapid detection plays a pivotal role in improving patient outcomes, and efficient management of diseases relies on timely diagnostics. High-throughput detection techniques enable the simultaneous analysis of large numbers of samples, which makes the diagnostic process faster and more efficient. Antibody-based immunoassays, which rely on the principle of antigen-specific binding of antibodies with high selectivity, have been widely used for the sensitive and accurate quantification of target antigens. Immunoassays leverage the high affinity and specificity of antibodies to detect even trace amounts of specific biomarkers, making them essential tools in diagnostics, including the detection of cancer biomarkers, such as programmed cell death ligand 1 (PDL1) and human epithelial growth factor receptor 2 (HER2) [1,2,3,4].

The enzyme-linked immunosorbent assay (ELISA) is the most commonly used immunoassay platform that employs antibodies for detecting antigens [5]. Direct ELISA uses enzyme-conjugated antibodies against the antigen, and the enzyme catalyzes a substrate reaction, resulting in an increase in absorbance [6]. Indirect ELISA uses a pair of antibodies: a primary antibody against the antigen and an enzyme-conjugated secondary antibody that recognizes the primary antibody. Various enzyme-conjugated universal antibodies that recognize the Fc region of primary antibodies and with a peptide tag attached to the primary antibody are available. If an enzyme-conjugated secondary antibody is not available, a secondary antibody and an enzyme-conjugated tertiary antibody can be used. However, the need for secondary and/or tertiary antibodies for indirect ELISA is not cost-effective, and the additional reaction and washing steps are time-consuming. Although direct ELISA offers the advantages of shorter assay time and greater convenience due to the absence of secondary or tertiary antibody reactions, enzyme-conjugated antibodies are sometimes not commercially available. Moreover, in-house generation of enzyme-conjugated antibodies often results in low conjugation efficiency [7,8,9]. This process can be inefficient and resource-intensive, leading to higher costs and reduced overall effectiveness. Additionally, if the purification step is eliminated to reduce loss and improve cost efficiency—by not performing the bead assay or size-exclusion chromatography—and non-labeled excess enzyme is mixed with the enzyme-conjugated antibody-containing sample, the enzyme can non-specifically attach to the well or antibody because of its large size [9]. Using ELISA for detecting PDL1 or HER2 typically requires approximately 20 h and involves several assay steps, making it time-consuming and labor-intensive [10,11,12,13]. The fluorogenic enzyme-linked immunosorbent assay (FLISA), an immunoassay that uses a fluorescent substrate instead of the horseradish peroxidase or alkaline phosphatase typically used in ELISA to increase sensitivity, requires approximately 21 h for PDL1 detection [14].

Fluorescence-linked immunosorbent assay (FLISA) has emerged as an alternative immunoassay method [15,16,17,18,19,20]. By conjugating a fluorescent dye to an antibody and using the dye as a probe, direct FLISA can be performed. The fluorescent dye is small, allowing excess dye to be eliminated during the washing step after fluorescent antibody incubation, resulting in negligible background signals. This differs from the effect of excess enzyme in direct ELISA, which can lead to higher background signals due to the larger size of enzyme. The small size of the fluorescent dye in FLISA allows for more efficient removal and reduces the chances of non-specific binding. Additionally, FLISA, which detects fluorescent signals, is more sensitive than ELISA, which uses absorbance signals. Furthermore, as there is a broader range of fluorescence colors available compared to enzyme products, multi-color assays for detecting multiple antigens are potentially feasible. Typically, direct FLISA involves three main steps: immobilizing the antigen in a 96-well plate at 4 °C overnight, blocking the well for 1–2 h with a blocking solution, such as bovine serum albumin (BSA)- or skim milk-containing antigen-dissolving buffer, or commercially available specialized blocking solution, and incubating the well with a fluorescent antibody for 1–2 h. After washing the well for a few minutes, the fluorescence intensity of each well is measured using a fluorescent scanner. Although commercially pre-coated plates are available, self-immobilization of antigens onto a plate offers advantages such as lower cost and more flexible antigen concentration. The total assay time for usual direct FLISA is over 20 h, which is time-consuming and involves long waiting periods [17,21,22]. This extended duration limits the practical application of FLISA in research and clinical fields that require rapid and efficient results for timely decision-making.

In this study, we established a rapid FLISA platform that reduces the fluorescent immunoassay time to a faster duration, making it comparable to the 23 h required by standard FLISA. We used single chain fragment variable regions (scFvs) against PDL1 and HER2 as model antibodies to optimize each step of the FLISA process, including antigen immobilization, blocking, and antibody reaction.

## 2. Materials and Methods

### 2.1. Materials

A 96-well black Maxi-plate was obtained from SPL (Gyeonggi-do, Korea). HER2 and mouse PDL1 proteins were obtained from Sino Biological (Beijing, China). NHS ester-conjugated TAMRA 6-isomer was obtained from Lumiprobe (Cockeysville, MD, USA). Guanidine hydrochloride was obtained from Daejung (Gyeonggi-do, Korea). Dithiothreitol (DTT) was obtained from LPS Solution (Daejeon, Korea). Other chemicals and reagents, unless otherwise indicated, were sourced from Sigma-Aldrich Korea (Seoul, Korea).

### 2.2. Expression of scFvs

Anti-PDL1 scFv was produced according to our previous study [23]. Briefly, SHuffle T7 Express LysY cells were transformed with pSrtCys::aPDL1scFv and cultured in 100 mL of LBA medium (LB medium containing 100 μg/mL ampicillin). When the OD600 reached 1.0, 1 mM isopropylthio-β-galactopyranoside (IPTG) was added, and the culture was incubated for an additional 16 h at 30 °C. After centrifugation, the pellet was sonicated, and the supernatant was purified using TALON resin. The eluent was subjected to ultrafiltration (3 kDa cut-off) and equilibrated with PBS. Anti-HER2 scFv was produced according to our previous study [24]. Briefly, BL21 (DE3) cells were transformed with pCold::HL-His and cultured in 100 mL of LBA medium. When the OD600 reached 1.2, 0.2 mM IPTG, 0.5% Triton X-100, and 0.05% Tween 20 were added, and the culture was incubated for an additional 72 h at 16 °C. Purification and ultrafiltration were performed using the same method as for the anti-PDL1 scFv.

### 2.3. Fluorescent Labeling

Fluorescent dye conjugation to scFv was performed according to our previous study [24]. Briefly, 300 μg of scFv in PBS was mixed with 10 molar excess of NHS ester-conjugated TAMRA and incubated in the dark at RT for 2 h. After labeling, 10 mM hydroxylamine was added to stop the reaction.

### 2.4. FLISA

100 μL of serially diluted PDL1 or HER2 protein was immobilized on a 96-well black Maxi-plate at 4 °C for 20 h, 37 °C for 4 h, 37 °C for 2 h, 37 °C for 1 h, or 37 °C for 10 min. After removing the solution by tapping the inverted plate, 400 μL of blocking buffer (PBS with 3% skim milk and 0.1% Tween 20) was added to the wells and incubated at RT for 2 h or 37 °C for 30 min. After removing the solution by tapping the inverted plate, the wells were washed once with 400 μL of PBST (PBS with 0.1% Tween 20). Subsequently, 1000 ng fluorescent anti-PDL1 or HER2 scFv in PBST was added to the wells and incubated at RT for 1 h, 37 °C for 30 min, or 37 °C for 10 min. After washing three times with 400 μL of PBST, the fluorescence intensity was measured using a fluorescence microplate reader (TECAN, Männedorf, Switzerland) with an excitation wavelength of 516 nm. The dose–response curves were fitted to a four-parameter equation, y = a + (b − a)/(1 + (c/x)^d), using GraphPad Prism software (version 10.4.1). In the equation, a, b, c, and d indicate the fluorescence intensity (F.I.) in the absence of the antigen, F.I. in the presence of the maximum antigen concentration, the amount of antigen at which the response is half-maximal (EC50), and the Hill slope, respectively. The LOD value, corresponding to the amount of antigen at which the response is 10% of the maximum (EC10), was calculated using the following equation:

EC_10_ = ((1/9)^(1/Hill slope)) × EC_50_ [25].

## 3. Results and Discussion

**Figure 1:**
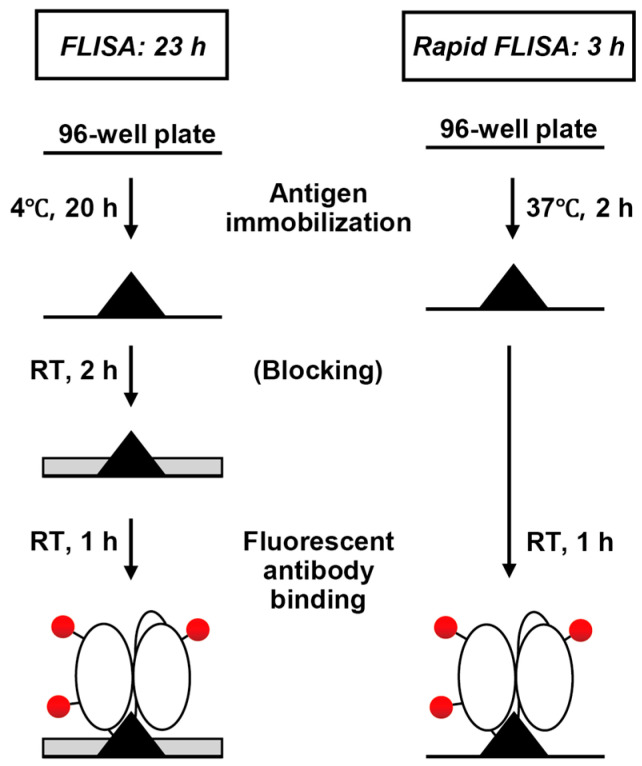
Schematic representation of the fluorescence-linked immunosorbent assay (FLISA) and rapid FLISA methods. Optimization of FLISA was performed in this study by adjusting the reaction time of each step. RT indicates room temperature.

The direct FLISA method primarily involves three steps: antigen immobilization, blocking, and fluorescent scFv binding. We optimized each of these steps to shorten the overall process (Figure 1).

**Figure 2:**
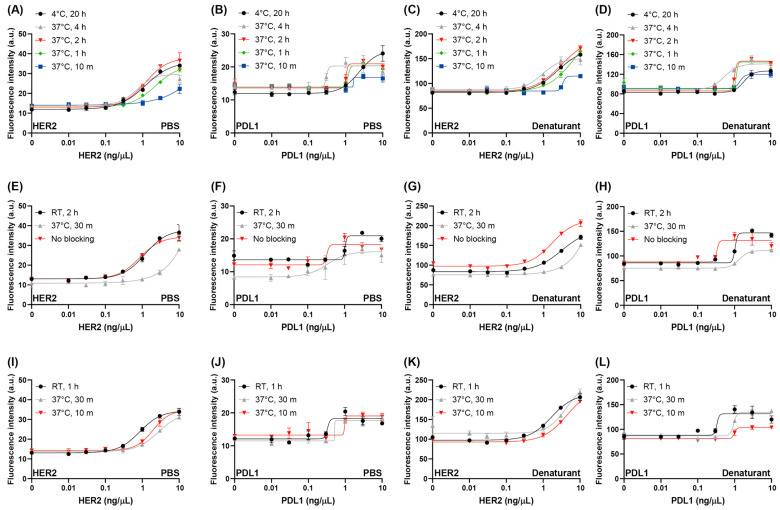
(**A**–**D**) Titration curves with different antigen immobilization conditions. (**E**–**H**) Titration curves with different blocking conditions. (**I**–**L**) Titration curves with different fluorescent antibody reaction conditions. (**A**,**E**,**I**) Fluorescence response in the presence of phosphate-buffered saline (PBS) for human epithelial growth factor receptor 2 (HER2) detection. (**B**,**F**,**J**) Fluorescence response in the presence of PBS for programmed cell death ligand 1 (PDL1) detection. (**C**,**G**,**K**) Fluorescence response in the presence of denaturant (7 M guanidine hydrochloride with 100 mM dithiothreitol in distilled water) for HER2 detection. (**D**,**H**,**L**) Fluorescence response in the presence of denaturant for PDL1 detection. Fluorescence intensity at 580 nm in the presence of the indicated antigen concentration was plotted in each titration curve. Error bars represent ±1 SD (*n* = 3). SD = standard deviation. RT indicates room temperature.

Typically, antigen immobilization for ELISA is performed at 4 °C overnight. However, our previous study demonstrated that the maximum immobilization efficiency for detecting TNFa, PDL1, and cell immobilization is influenced by both temperature and duration [14,16]. Based on these findings, we tested different immobilization conditions, following typical steps that included blocking at RT for 2 h and incubating with the fluorescent antibody at RT for 1 h (Figure 2A–D). After washing off the unbound fluorescent antibody and measuring the fluorescence intensity, we observed an increase in intensity in an antigen concentration-dependent manner. When immobilizing the HER2 protein as the antigen, the limit of detection (LOD) was 0.20 ng/μL at 4 °C for 20 h, which was similar to the value observed with immobilization at 37 °C for 4 or 2 h. However, when immobilization was performed at 37 °C for 1 h, the response decreased, and it was further reduced when the immobilization time was shortened to 10 min. When PDL1 was used as the antigen and anti-PDL1 scFv as the antibody, the LOD was at 4 °C for 20 h was 0.66 ng/μL. The response was similar at 37 °C for 4, 2, or 1 h but decreased at 37 °C for 10 min. These results indicate that antigen immobilization efficiency was reduced when the immobilization time was shortened at higher temperatures. However, the response at 4 °C for 20 h was maintained at higher temperatures when the immobilization time was longer than 2 h for PDL1 and 1 h for HER2. Based on these findings, we chose to immobilize the antigen at 37 °C for 2 h. Although the signal in the PDL1 assay system was slightly higher at 37 °C 4 h compared to 2 h, we selected the 2 h condition to reduce the overall assay time, which aligns with the objective of this study.

We measured the fluorescence intensity in the presence of a denaturant because the TAMRA dye, a rhodamine dye with aromatic rings, can have its fluorescence quenched by the intrinsic aromatic amino acids of proteins, such as tryptophan and tyrosine [26]. When the distance between the dye and the aromatic amino acid is reduced by forming a dye-conjugated protein complex, hydrophobic interactions or π-π stacking interactions between the aromatic rings of the dye and the aromatic amino acid stabilize the excited state electrons of the dye. This leads to photo-induced electron transfer (PeT) from the aromatic amino acid to the dye, resulting in quenching [27,28]. This quenching effect results in decreased fluorescence intensity compared to non-quenched. When the denaturant was added, the three-dimensional structure of protein was disrupted, causing the amino acids to unfold into a linear form, which led to the de-quenching of the dye [29]. In other words, we hypothesized that if some of the dye conjugated to the antibody was quenched, the response would improve upon the addition of a denaturant.

We tested this approach to further enhance the response. After discarding the PBS and adding the denaturant to the same well, we measured the fluorescence intensity. The responses for both HER2 and PDL1 under various immobilization conditions were similar to those observed in the presence of PBS. For the PDL1 assay, the response at 37 °C for 4, 2, or 1 h was similar to that at 4 °C for 20 h, while the response at 37 °C for 10 min showed a decrease. In the HER2 assay system, the response at 37 °C for 4 h was slightly higher than at other conditions. These results confirmed our decision to proceed with the next step using the immobilization condition fixed at 37 °C for 2 h. The maximum fold increase in the PDL1 assay in the presence of PBS and denaturant was similar: 1.95 ± 0.19, 1.31 ± 0.09, 1.35 ± 0.06, 1.33 ± 0.05, and 1.14 ± 0.08-fold at 20 h, 4 h, 2 h, 1 h, and 10 min, respectively, in PBS, and 1.48 ± 0.04, 1.50 ± 0.09, 1.69 ± 0.05, 1.38 ± 0.04, and 1.26 ± 0.06-fold at 20 h, 4 h, 2 h, 1 h, and 10 min, respectively, in the presence of denaturant. These results suggest that there was no quenched dye and that the directions of the conjugated dyes were facing outward of the protein in the presence of PBS. After adding the denaturant, the protein was linearized, and the dyes exhibited independent orientations without quenching. In the HER2 assay, the maximum fold increase in the presence of PBS was 2.87 ± 0.06, 2.03 ± 0.13, 2.81 ± 0.31, 2.40 ± 0.08, and 1.63 ± 0.17-fold at 20 h, 4 h, 2 h, 1 h, and 10 min, respectively. In the presence of the denaturant, the maximum fold increase was 1.95 ± 0.03, 1.66 ± 0.12, 1.96 ± 0.06, 1.86 ± 0.05, and 1.31 ± 0.03-fold at 20 h, 4 h, 2 h, 1 h, and 10 min, respectively. Unexpectedly, the responses for the denatured antibodies were lower than those for the native antibodies. We suggest that after denaturation, the distance between the dyes decreased, leading to the formation of an H-dimer, which resulted in decreased signals [30]. To further confirm why the signal in the HER2 system was lower in the presence of denaturant compared to PBS, we decided to proceed to the next step without further investigation, as we considered the denaturant assay as an optional approach to improving the responses.

Next, the antigen was immobilized at 37 °C for 2 h, and the blocking condition was optimized (Figure 2E–H). Typically, blocking in immunoassays is performed at RT or 37 °C for 1–2 h. When we added the blocking solution (PBS with 3% BSA) and incubated at 37 °C for 0.5 h, the response decreased, while the initial signal in the absence of the antigen remained similar. This suggested that over-blocking at a higher temperature interfered with fluorescent antibody binding. When we skipped the blocking step and simply rinsed the well with PBS once, followed by the addition of the fluorescent antibody, the signal was similar to or slightly improved compared to the traditional blocking condition (RT, 2 h). This indicates that blocking is not a necessary step for this FLISA system. Generally, the blocking step in immunoassays is included to prevent non-specific binding of reagents to the well surface [31,32]. In this study, we added 0.05% Tween20 to both the fluorescent antibody solution and the washing buffer, which may have prevented non-specific binding of free dye that did not bind to the scFv but remained in the sample [33]. The effectiveness of the no-blocking strategy was also demonstrated by the response after adding the denaturant.

Based on these results, we optimized the fluorescent antibody reaction conditions with the following fixed parameters: antigen immobilization at 37 °C for 2 h, followed by a PBS rinse without a blocking step (Figure 2I–L). Typically, antibody binding in immunoassays is performed at RT for 1 h. We shortened the reaction time but increased the temperature to 37 °C. As a result, the responses under the shortened conditions were slightly decreased, thus we determined the optimal fluorescent scFv reaction condition to be RT for 1 h.

**Figure 3:**
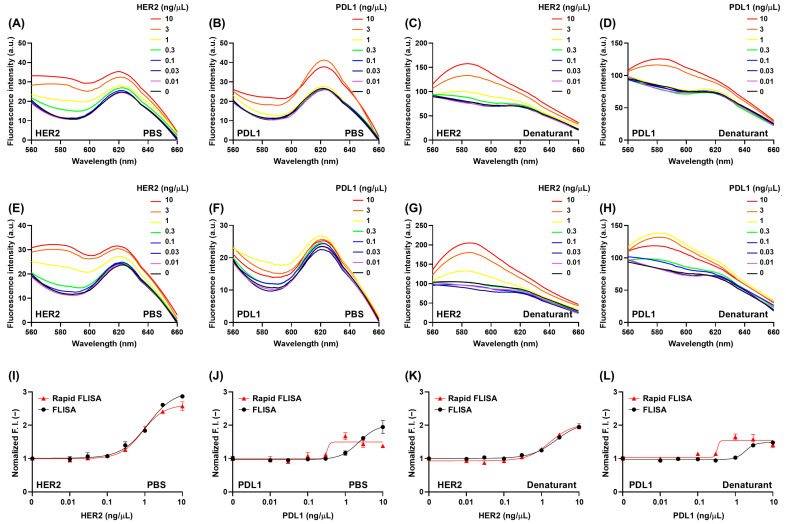
(**A**–**D**) Fluorescence spectra of fluorescence-linked immunosorbent assay (FLISA) (antigen immobilization for 20 h, blocking for 2 h, and fluorescent antibody binding for 1 h) in the presence of phosphate-buffered saline (PBS) or denaturant (7 M guanidine hydrochloride with 100 mM dithiothreitol in distilled water) for programmed cell death ligand 1 (PDL1) or human epithelial growth factor receptor 2 (HER2) detection. (**E**–**H**) Fluorescence spectra of rapid FLISA (antigen immobilization for 2 h and fluorescent antibody binding for 1 h) in the presence of PBS or denaturant for PDL1 or HER2 detection. (**I**–**L**) Normalized fluorescence intensity of FLISA or rapid FLISA in the presence of PBS or denaturant for PDL1 or HER2 detection. Normalized fluorescence intensity (F.I.) was calculated by dividing the F.I. of each sample with the indicated antigen concentration by the F.I. of the sample without antigen. Error bars represent ±1 SD (*n* = 3). SD = standard deviation.

When we compared the fluorescent spectra and responses between the optimized and non-optimal conditions, the titration curve for detecting HER2 showed similar signals in both PBS and denaturant solutions (Figure 3 and Figure S1). The LOD values for FLISA and rapid FLISA with anti-HER2 scFv in PBS were 200 and 198 pg/μL, respectively. The LOD values for FLISA and rapid FLISA in denaturant were 450 and 368 pg/μL, respectively. The LOD values for FLISA and rapid FLISA with anti-PDL1 scFv in PBS were 660 and 274 pg/μL, respectively. The LOD values for FLISA and rapid FLISA with anti-PDL1 scFv in denaturant were 975 and 282 pg/μL, respectively. The titration curve for detecting PDL1 in both PBS and denaturant showed improved responses in the updated method compared to the unoptimized method. In particular, the difference in signal at 1 ng/μL PDL1 was noticeable, indicating that the FLISA assay under the optimized conditions exhibited higher sensitivity. Specifically, the titration curves in both PBS and the denaturant showed similar or increased responses under the optimized conditions, and the overall assay time was significantly reduced from 23 h to 3 h. The signal in the presence of HER2 in PBS increased up to 2.57 ± 0.13-fold in rapid FLISA and 2.87 ± 0.06-fold in FLISA, respectively. The signal in the presence of HER2 in denaturant increased up to 1.97 ± 0.08-fold in the rapid FLISA and 1.95 ± 0.03-fold in FLISA, respectively. The signal in the presence of PDL1 in PBS increased up to 1.38 ± 0.05- and 1.95 ± 0.19-fold in rapid FLISA and FLISA, respectively. The signal in the presence of PDL1 in denaturant increased up to 1.41 ± 0.09- and 1.48 ± 0.04-fold in rapid FLISA and FLISA, respectively. Overall, the signal in the denaturant decreased, likely due to the previously mentioned formation of H-dimers.

Moreover, we performed rapid FLISA using variable cancer-related antigens stored in our laboratory, such as HER2, PDL1, matrix metalloproteinase9 (MMP9), S100 calcium-binding protein A8 (S100A8), S100 calcium-binding protein A9 (S100A9), and tumor necrosis factor alpha (TNFα), which are cancer biomarkers, as well as cytotoxic T-lymphocyte associated protein 4 (CTLA4), an immune checkpoint (Figure S2). When we added fluorescent anti-HER2 scFv, a high signal was detected from the HER2-immobilized well, whereas other antigens showed negligible signals. When we added fluorescent anti-PDL1 scFv, a high signal was observed from the PDL1-immobilized well, while other antigens, except for S100A9, showed negligible signals. When we added anti-PDL1 scFv to a S100A9-immobilized well and measured the fluorescence intensity in the presence of PBS, the signal was similar to that observed with the PDL1-immobilized well. These results indicate the specificity of this FLISA system. Performing FLISA in both PBS and denaturing conditions is preferred for obtaining a clear determination of analytical specificity, especially when S100A9 is present in the sample for PDL1 detection. To summarize, we recommend the FLISA method with the optimized protocol, using PBS as the detection solution, to reduce overall assay time while maintaining or improving sensitivity. The total reaction time is 3 h, with minimal hands-on time and primarily waiting periods. The process involves adding the antigen to the well, incubating the plate for 2 h, discarding the solution, adding PBS, discarding again, adding the fluorescent antibody, and incubating the plate for 1 h. After discarding the solution, the fluorescent spectrum is measured. The 3 h assay time is mainly composed of waiting periods. Additionally, measuring the fluorescent spectrum and plotting the fluorescence intensity at the TAMRA wavelength (580 nm) is straightforward and easy to interpret.

This study is the first to establish a rapid FLISA system for immunoassay targeting PDL1 and HER2 antigens, providing exact and direct comparison data under various assay conditions. This rapid and convenient assay method offers significant advantages for use in various immunoassays. Additionally, rapid FLISA requires only a fluorescent antibody, whereas other immunoassays, such as ELISA, typically require a primary antibody, a secondary, and/or a tertiary antibody, with one of these antibodies conjugated to an enzyme. Moreover, the enzyme requires both a substrate and a stop solution. In contrast, the rapid FLISA developed in this study only requires a fluorescent antibody, making it a more cost-effective alternative by reducing reagent requirements

This makes rapid FLISA an ideal choice for routine diagnostics, screening, and large-scale studies. The improved sensitivity, coupled with the ease of use and minimal hands-on time, provides a reliable and efficient alternative to traditional immunoassay methods. To further apply this system for cancer diagnosis, PDL1- and HER2-overexpressing cell lines can be immobilized on the plate, and the response can be compared with that of non-cancer cell lines. Following these in vitro cellular tests, rapid FLISA can be performed by immobilizing cells from patient samples for in vivo testing. We plan to conduct these assays to confirm the applicability of this rapid FLISA system for the actual diagnosis of cancer in subsequent studies.

## Data Availability

The data that support the findings of this study are available from the corresponding author.

## Supplementary Materials

The following supporting information can be downloaded at: https://www.mdpi.com/article/10.3390/cimb47010062/s1.
