# Supplementary material for: Establishment of a Rapid and Convenient Fluoroimmunoassay Platform Using Antibodies Against PDL1 and HER2
Source: Curr Issues Mol Biol. 2025 Jan 17;47(1):62. doi: 10.3390/cimb47010062 (PMC11763867; doi:10.3390/cimb47010062)
Supplement: Supplementary file 1 [file cimb-47-00062-s001.zip › cimb-3377945-supplementary.pdf]

Establishment of a Rapid and Convenient Fluoroimmunoassay Platform Using Antibodies Against PDL1 and HER2

Ji Eun Choi [1], Hanool Yun [2] and Hee-Jin Jeong [2,*]

[1] Department of Pathology, Chungnam National University Sejong Hospital, Sejong 30099, Republic of Korea
[2] Department of Biological and Chemical Engineering, Hongik University, Sejong 30016, Republic of Korea
* Correspondence: heejinjeong@hongik.ac.kr

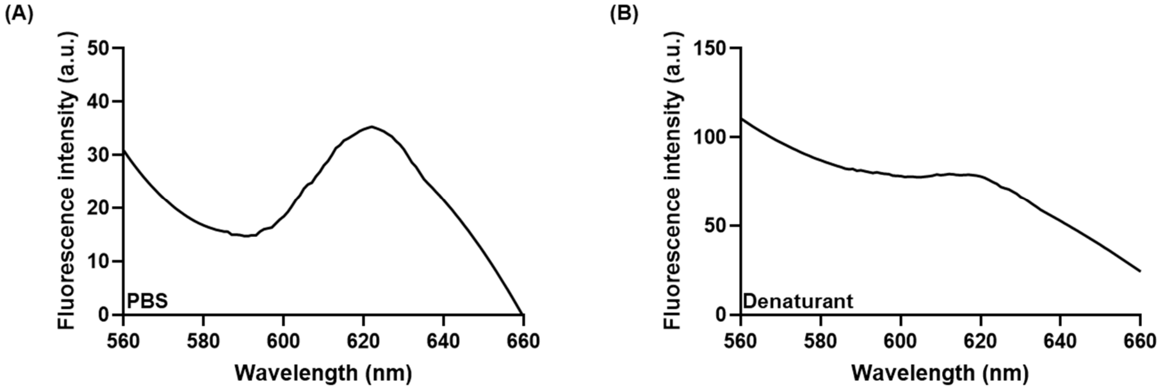

Figure S1. (A) Fluorescence spectrum of phosphate-buffered saline (PBS), (B) Fluorescence spectrum of denaturant (7 M guanidine hydrochloride with 100 mM dithiothreitol in distilled water). A volume of 100 μL of PBS or denaturant was added to the well of a 96-well plate, and the fluorescent intensity was measured using a fluorescent spectrometer.

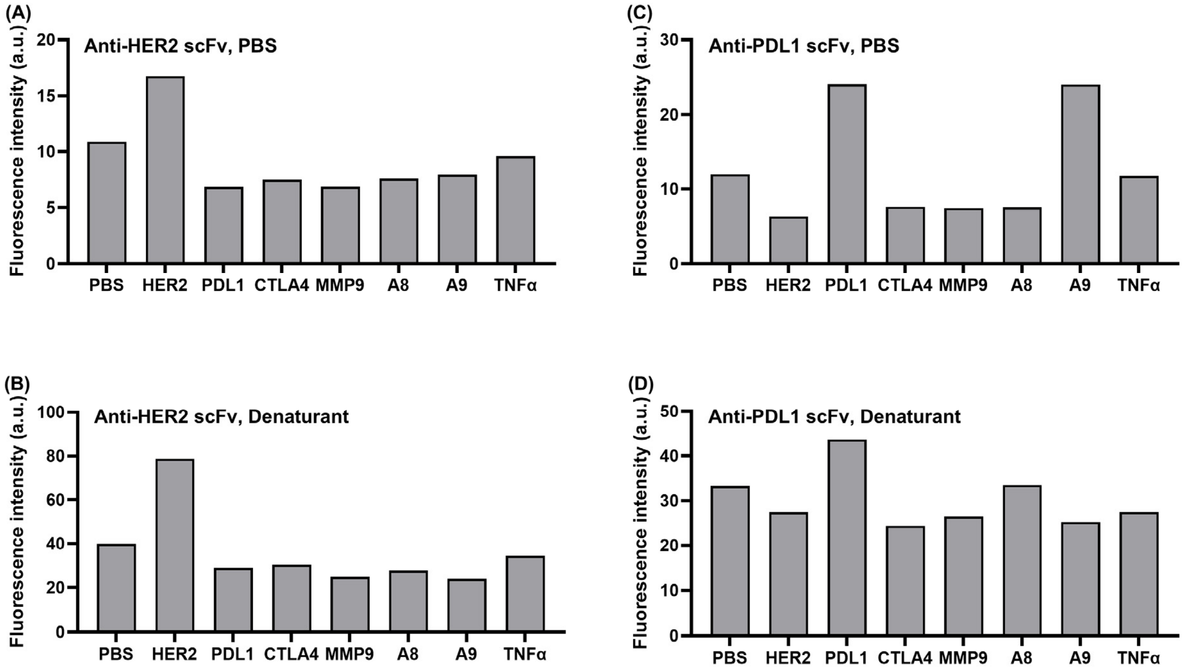

Figure S2. (A, B) Rapid FLISA response using fluorescent anti- human epithelial growth factor receptor 2 (HER2) scFv (A) and fluorescent anti-programmed cell death ligand 1 (PDL1) scFv (B) in the presence of PBS. (C, D) Rapid FLISA response using fluorescent anti-HER2 scFv (C) and fluorescent anti- PDL1 scFv (D) in the presence of PBS. Various antigens, including phosphate-buffered saline (PBS), HER2, PDL1, matrix metalloproteinase9 (MMP9), S100 calcium-binding protein A8 (S100A8), S100 calcium-binding protein A9 (S100A9), and tumor necrosis factor alpha (TNFα), and cytotoxic T-lymphocyte associated protein 4 (CTLA4) were immobilized in each well at 10 ng/μL, and rapid FLISA was performed. Fluorescence intensity at 580 nm in the presence of the indicated antigen was plotted.
